# Serine Supports IL-1β Production in Macrophages Through mTOR Signaling

**DOI:** 10.3389/fimmu.2020.01866

**Published:** 2020-08-27

**Authors:** Siyuan Chen, Yaoyao Xia, Fang He, Jian Fu, Zhongquan Xin, Baichuan Deng, Liuqin He, Xihong Zhou, Wenkai Ren

**Affiliations:** ^1^State Key Laboratory for Conservation and Utilization of Subtropical Agro-Bioresources, Guangdong Laboratory of Lingnan Modern Agriculture, Guangdong Provincial Key Laboratory of Animal Nutrition Control, National Engineering Research Center for Breeding Swine Industry, College of Animal Science, South China Agricultural University, Guangzhou, China; ^2^College of Animal Science and Technology, Southwest University, Chongqing, China; ^3^Hunan International Joint Laboratory of Animal Intestinal Ecology and Health, Laboratory of Animal Nutrition and Human Health, College of Life Sciences, Hunan Normal University, Changsha, China; ^4^Key Laboratory of Agro-ecological Processes in Subtropical Region, Hunan Provincial Key Laboratory of Animal Nutritional Physiology and Metabolic Process, National Engineering Laboratory for Pollution Control and Waste Utilization in Livestock and Poultry Production, Institute of Subtropical Agriculture, Chinese Academy of Sciences, Changsha, China

**Keywords:** macrophage, mTOR, IL-1β, inflammation, serine

## Abstract

Intracellular metabolic programs tightly regulate the functions of macrophages, and previous studies have shown that serine mainly shapes the macrophage function via one-carbon metabolism. However, it is unknown whether serine modulates the macrophage function independent of one-carbon metabolism. Here, we find that serine deprivation lowers interleukin (IL)-1β production and inflammasome activation, as well as reprograms the transcriptomic and metabolic profile in M1 macrophages. Intriguingly, supplementation of formate, glycine, dNTPs, and glucose cannot rescue the production of IL-1β from serine-deprived macrophages. Mechanistically, serine deprivation inhibits macrophage IL-1β production through inhibition of mechanistic target of rapamycin (mTOR) signaling. Of note, the macrophages from mice feeding serine-free diet have lower IL-1β production, and these mice also show less inflammation after LPS challenge. Collectively, our data highlight a new regulatory mechanism for serine to modulate the macrophage function.

## Introduction

Macrophages are essential components for resisting pathogen infection, repairing damaged tissue, and maintaining immune homeostasis (1, 2). Under stimulation with LPS and/or IFN-γ, macrophages polarize into M1 macrophages to show pro-inflammatory phenotype and resist bacterial infection through the production of interleukin (IL)-1β, tumor necrosis factor (TNF)-α, and nitric oxidate (NO) (2, 3). However, M2 macrophages induced by IL-4 and/or IL-13 show anti-inflammatory phenotype and repair damaged tissue (4, 5). As deregulated phenotypic switch in macrophages highly affects host immune homeostasis; it is of great importance to fully understand the regulatory mechanisms that orchestrate M1/M2 macrophage polarization.

The fine-tuning of macrophage phenotypes includes intracellular signaling cascades, epigenetic rewiring, and metabolic programming (6). For example, M1 macrophages rely on aerobic glycolysis, pentose phosphate pathway (PPP), and fatty acid synthesis (7). In contrast, the metabolic characteristics in M2 macrophages include mitochondrial oxidative phosphorylation (OXPHOS) and fatty acid oxidation, and an intact tricarboxylic acid (TCA) cycle (8). In addition to these metabolic differences, M1 macrophages and M2 macrophages also differ in amino acid metabolism. For example, arginine is converted to NO by inducible NO synthase (iNOS) in M1 macrophages, while in M2 macrophages arginine is converted to polyamine by arginase (9, 10). Through UDP-N-acetylglucosamine and α-ketoglutarate, glutamine induces polarization of M2 macrophages, while glutamine promotes M1 macrophage polarization through succinate from glutamine-dependent anerplerosis or the γ-aminobutyric acid shunt (8, 11). Of note, there is growing interest in serine metabolism in fate decision of macrophages.

Serine is a non-toxic non-essential amino acid and has diverse physiological functions (12, 13), including the immunoregulatory actions (14–16). Through one-carbon metabolism in macrophages, serine is critical for the generation of phospholipid, biosynthesis of purine and thymidine, and production of methyl donor of S-adenosyl-methionine (SAM) and cellular glutathione (17–21). For example, serine regulates macrophage IL-1β production through serine-glycine-glutathione axis (22). However, it is interesting to explore whether serine affects macrophage functions through other mechanisms.

The mechanistic target of rapamycin (mTOR) is a nutrient-sensing kinase that integrates input from amino acids, growth factors, and the energy status of the cell. It has been well-accepted that the mTOR signaling pathway not only favors in the differentiation and activation of macrophages (23, 24), but also benefits macrophage polarization (chiefly M1 macrophage) and metabolism (25–27). Here, we find that serine affects production of IL-1β from macrophages through the mTOR signaling. These results highlight new regulatory mechanism by which serine tailors M1 macrophage polarization, and suggest that targeting serine-mTOR axis would be an attractive strategy for modulating the progression of macrophage-associated diseases.

## Materials and Methods

### Mice

Female ICR mice (Institute of Cancer Research; 6–8 weeks) were obtained from SLAC Laboratory Animal Center (Changsha, China) and housed in individually ventilated, pathogen-free cages (temperature at 20–30°C, relative humidity at 50–60%, lighting cycle at 12 h/day) with free access to food and water. All experimental protocols were approved by and under the conduction of the Laboratory Animal Ethical Commission of the South China Agricultural University.

### Antibodies

Antibodies against AIM2 (ab180665), Caspase 1 (ab179515), NLRC4 (ab201792), and NLRP3 (ab214185) were purchased from Abcam (Cambridge, UK). Antibodies against p-IκB (Sc-8404), IKK (Sc-7607), and p-IKK (Sc-21660) were purchased from Santa Cruz Biotechnology, Inc. (Dallas, Texas, USA). Antibodies against HIF-1α (14179s), IL-1β (12426s), mTOR (2972s), and p-mTOR (5536s) were purchased from Cell Signaling Technology (Danvers, MA, USA). Antibodies against IκB (51066-1-AP), p65 (10745-1-AP), and β-actin (60008-1-Ig) were purchased from Proteintech (Rosemont, IL, USA). Antibody against NALP1(PA5-17275) was purchased from Thermo Fisher (Danvers, MA, USA). Antibody against p-P65 (bs-0982R) was purchased from Bioss (Beijing, China).

### Isolation of Thioglycolate-Elicited Peritoneal Macrophages (TGPMs)

Isolation of the peritoneal macrophages from mice was conducted as described in previous studies with some minor modifications (28). Briefly, 3 days after the injection of 4% thioglycolate, macrophages were flushed from peritoneal cavity with cold Dulbecco's modified Eagle's medium (DMEM) (Gibco), and cultured in complete DMEM medium (10% FBS, 1% penicillin/streptomycin). After adherent purification, macrophages were treated as indicated.

### Treatments in Macrophages

Macrophages were stimulated with LPS (1 μg/ml, Invitrogen) plus IFN-γ (20 ng/ml, Peprotech) and cultured with medium which deprived serine completely or supplemented with serine. Treatments (like formate, glycine, dNTPs, glucose or sodium pyruvate, rapamycin, MHY1485 or leucine) were supplemented in above medium as indicated concentration (21, 29, 30).

### Treatments in Cell Line

ANA.1 cells were cultured in RPMI 1640 (Gibco) supplemented with 10% FBS and 1% penicillin/streptomycin at 37°C with 5% CO2. Cells were stimulated with LPS (1 μg/ml, Invitrogen) plus IFN-γ (20 ng/ml, Peprotech) for 15 h. Serine was deprived in the medium as indicated.

### Intracellular Transfection of Metabolites

The macrophages were isolated and purified as described above. For pyruvate or glucose delivery, Optimem media [basal DMEM medium containing pyruvate (10 mM) alone or combined with glucose (5 mM), and 5 μl/ml of FuGENE® HD Transfection reagent (Promega, Madison, Wisconsin, USA)] was added to macrophages for 45 min at 37°C in 5% CO_2_. Subsequently, the permeabilization buffer was removed and macrophages were cultured with normal complete DMEM medium for 24 h (31). Finally, the above medium was replaced with serine-deprived medium and macrophages were stimulated as indicated.

### LPS Challenge in Mice

Female ICR mice (3 weeks) were divided randomly into two groups (*n* = 10 in control group for normal diet, and *n* = 10 in treatment group for serine-free diet) and permitted *ad libtium* access to feed and water for 4 weeks. Then, the serum was collected and TGPMs were isolated for further analysis. For the sepsis model, mice were challenged with LPS intraperitoneally (10 mg/kg body weight). At 15 h of LPS challenge, the lung, liver, jejunum and colon of each mouse were collected for further assays.

### Analysis of Glucose Uptake

The glucose uptake in macrophages was analyzed as described below. The TGPMs are stimulated with LPS plus IFN-γ for 15 h, and then the culture medium was removed and the cells were washed with PBS for 3–4 times. Subsequently, the new medium with 2-NBDG (a fluorescence probe to detect glucose uptake) (100 μM) was added for 20 min at 37°C with 5% CO_2_, followed by washing with PBS for 3–4 times and then the fluorescence intensity was recorded.

### Quantitative PCR

Total RNA of macrophages was extracted using Trizol (Invitrogen, USA) and then quantified with Nanodrop 2000. After the reverse transcription using Primer Script TM RT reagent Kit (Takara, Qingdao, China), quantitative PCR (qPCR) was conducted via SYBR Green on the QuantStudio 6 Real-Time PCR System (Thermo Fisher, America) as following: denaturation at 95°C for 10 min, amplification at 95°C for 15 s and 60°C for 1 min for 40 cycles. Each target gene was individually normalized to the reference gene β-actin by using the quantification method of 2^−ΔΔ*Ct*^. Primers used in this study were listed in Supplementary Table 1.

### Enzyme-Linked Immunosorbent Assay (ELISA)

The culture supernatants were harvested and centrifuged at 1,000 rpm for 5 min before analysis. The homogenated lung tissues or serum were centrifuged at 12,000 rpm for 10 min with the temperature of 4°C before analysis. Concentration of cytokines (IL-1β and TNF-α) in culture supernatants, as well as mouse tissues, and serum were analyzed with mouse ELISA kits (KE10002 and KE10003, Proteintech, Rosemont, IL 60018, USA) according to protocol from manufacturer.

### Western Blot

Western blot assay was performed as follows. Briefly, TGPMs were lysed in ice-cold RIPA for 10 min. The supernatant was used to measure the protein concentration by bicinchoninic acid (BCA) protein assay kits (Beyotime, China). After separating by 10% SDS-PAGE electrophoresis, proteins were transferred onto a PVDF membrane (BioRad, USA), and blocked with 5% BSA Tris-Tween-buffered saline buffer (TBST) for 1 h. Then the membranes were incubated with primary antibodies overnight at 4°C. Subsequently, the horseradish peroxidase (HRP)-conjugated secondary antibodies (anti-rabbit 1:6,000, Abcam; anti-mouse 1:5,000, proteintech; anti-goat 1:30,000, Santa.) were incubated for 90 min at 37°C. Finally, the membrane-developed blots were developed, observed and analyzed by using AlphaImager 2200 software (Alpha Innotech Corporation, CA, USA). Signal intensity was quantified and normalized to Actin protein abundance.

### Transcriptomic Analysis

TGPMs were treated as indicated and harvested for transcriptomic analysis in NovoGene (Beijing, China). Briefly, clean data were obtained by removing low quality reads from raw data and reference transcript. Differential expression analysis was performed in DESeq, and *p* < 0.05 was considered as the threshold for significantly differential expression. GO and KEGG pathway enrichment analysis of the DEGs were then performed. All the transcriptomic data have been deposited to NCBI's Gene Expression Omnibus (GEO) database with the accession number (PRJNA527189).

### Metabolites Analysis

To analyze the metabolites in macrophages, TGPMs (1^*^10^7^ cells/well) were treated as indicated and collected, and metabolites were quenched and extracted for metabolite quantification by gas chromatography-mass spectrometer (GC-MS).

### Seahorse Assay

After indicated treatments, changes in OCR (oxygen consumption rate) and ECAR (extracellular acidification) of macrophages were determined on a Seahorse XF24 Extracellular Flux Analyzer (Agilent Technologies). OCR assays were performed with 2 mM glutamine and 10 mM glucose and adjusted to pH 7.4, then following compounds were added in sequence: 100 μM oligomycin, 100 μM carbonyl cyanide 4-(trifluoromethoxy) phenylhydrazone (FCCP), and 50 μM rotenone plus antimycin A. ECAR assays were performed with 1 mM glucose, 1 mM oligomycin, and 1 mM 2-deoxy-glucose (2-DG).

### Tissue Histological Analysis

The fixed slices were dehydrated in graded ethanol, embedded in paraffin, sectioned, and stained with hematoxylin and eosin (H&E) for histopathological examinations or used for lung immunohistochemical staining of F4/80 as our recent study (14).

### Statistical Analyses

Data shown are the means ± SEM or SD. Data between two groups were analyzed by unpaired *t*-test (Prism 6.0) if the data were in Gaussian distribution and had equal variance, or by unpaired *t*-test with Welch's correction (Prism 6.0) if the data were in Gaussian distribution but with unequal variance, or by non-parametric test (Mann-Whitney *U*-test, Prism 6.0) if the data were not normally distributed. Data among more than two groups were analyzed by the one-way ANOVA followed by Dunnett multiple comparisons (Prism 6.0) if the data were in Gaussian distribution and had equal variance, or analyzed by Kruskal-Wallis followed by Dunn's multiple comparisons (Prism 6.0) if the data were not normally distributed. The Gaussian distribution of data was analyzed by D'Agostino-Pearson omnibus normality test (Prism 6.0) and Kolmogorov-Smirnov test (Prism 6.0). The variance of data was analyzed by homogeneity of variance test (SPSS 22.0) or Brown-Forsythe test (Prism 6.0). Differences with *p* < 0.05 were considered significant.

## Results

### Serine Deprivation Affects M1 Macrophage Function

**Figure 1:**
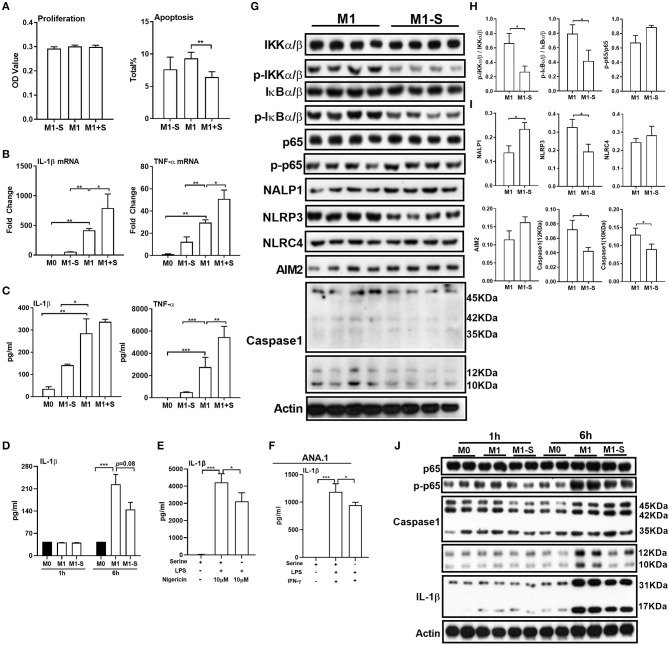
Serine deprivation influences M1 macrophage function. **(A)** The proliferation and apoptosis of TGPMs (*n* = 4–6). The total apoptosis was analyzed with flow cytometry analysis. Data are representative of three independent experiments. **(B)** mRNA expressions of IL-1β and TNF-α in TGPMs. Data are representative of two independent experiments with *n* = 4 per group and shown as the means ± SEM. **(C)** The secretion of IL-1β and TNF-α from TGPMs (*n* = 4). Data are representative of three independent experiments. **(D)** The secretion of IL-1β from TGPMs at 1 or 6 h post treatment. **(E)** The secretion of IL-1β from TGPMs in completed medium and serine deficiency medium (*n* = 4) with LPS (1μg/ml) for 11 h and then stimulated with nigericin (10 μM) for 1 h. **(F)** The secretion of IL-1β from ANA.1 macrophages stimulated with LPS (1μg /ml) plus IFN-γ (20 ng/ml) in completed medium and serine-deficient medium (*n* = 4). **(G–I)**. **(G)**: western bolt showing the protein abundance of IKKα/β, p-IKKα/β, IkBα/β, p-IkBα/β, p65, p-p65, NALP1, NLRP3, NLRC4, AIM2, and Caspase-1 in TGPMs; **(H,I)**: statistically analysis the activation of these pathways in different groups (*n* = 4). **(J)** Representative western bolt displaying time-dependent change of protein abundance of IL-1β, p65, p-p65, and Caspase-1 in TGPMs (*n* = 4). M0:thioglycolate-elicited peritoneal macrophages (TGPMs) without any treatment; M1-S: TGPMs were stimulated with LPS (1 μg/ml) plus IFN-γ (20 ng/ml) in serine deficiency medium; M1: TGPMs were stimulated with LPS (1 μg/ml) plus IFN-γ (20 ng/ml) in completed medium; M1+S: TGPMs were stimulated with LPS (1 μg/ml) plus IFN-γ (20 ng/ml) in medium with serine supplementation at dosage of 1.2 mM. Macrophages were stimulated with LPS plus IFN-γ for 15 h except indicated. Data were analyzed with one-way ANOVA **(A–G)** or unpaired *t*-test **(I,J)** and represented as means ± SD except indicated. *P < 0.05, **P < 0.01, ***P < 0.001.

To determine whether serine is critical for production of proinflammatory cytokines in M1 macrophages, we exposed TGPMs to LPS/IFN-γ for 15 h in media depleted of or supplemented with serine. Serine deprivation had little effect on proliferation and apoptosis of M1 macrophages, while serine supplementation suppressed apoptosis (Figure 1A). Serine deprivation lowered the mRNA expression of IL-1β and TNF-α in M1 macrophages, while serine supplementation boosted the expression of these genes (Figure 1B). Notably, serine deprivation significantly reduced the production of cytokines, especially IL-1β, from macrophages at 6 or 15 h post LPS/IFN-γ treatment (Figures 1C,D). Interestingly, serine deprivation also lowered the production of IL-1β from macrophages treated with LPS and nigericin (Figure 1E). Similar to TGPMs, serine was also necessary for the production of IL-1β from ANA.1 cells (Figure 1F). Considering M1 macrophage function involves in the activation of intracellular signaling pathways, like NF-κB and inflammasome (32, 33); thus, we analyzed the activation of these pathways. Serine deprivation remarkably blocked the activation of IKKα/β, IκBα/β, NLRP3, and Caspase-1 in M1 macrophages (Figures 1G,I), and even at 1 h post treatment for NF-κB and 6 h post treatment for Caspase-1 (Figure 1J). Together, these results indicate that serine is crucial for M1 macrophage function.

### Serine Deprivation Alters the Transcriptomic Profile of M1 Macrophages

**Figure 2:**
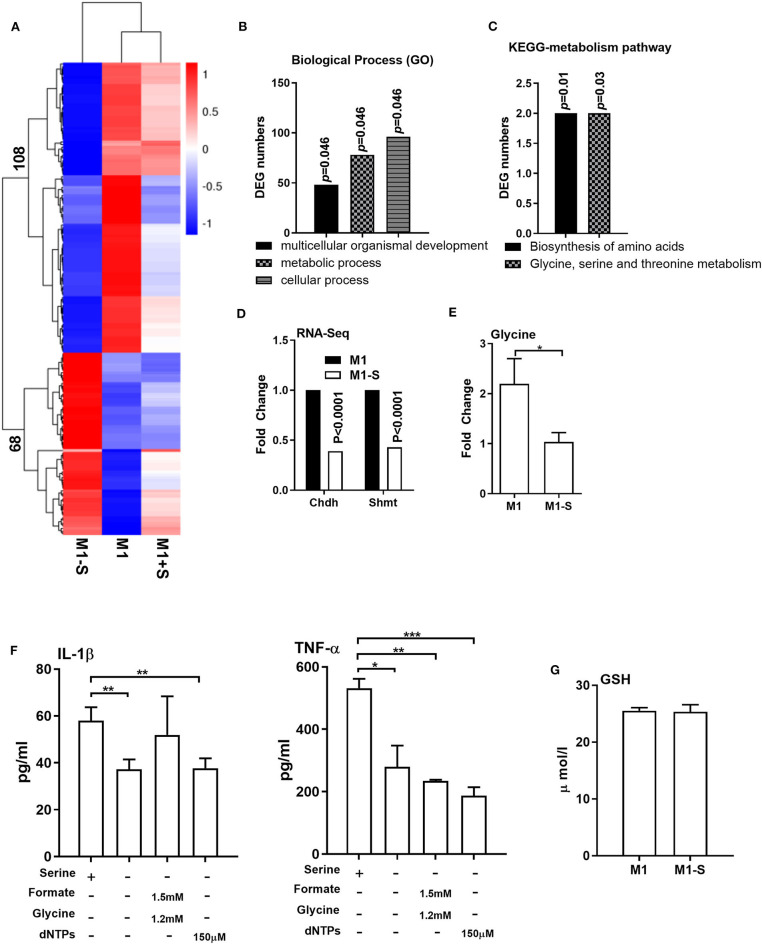
Serine deprivation alters the transcriptomic profile of M1 macrophages. **(A)** Heatmap analysis of up-regulated or down-regulated genes among different groups (*n* = 4). **(B)** GO analysis of DEG numbers which enriched in biological process between M1 and M1-S (*n* = 4). **(C)** The effect of serine deprivation on “Biosynthesis of amino acids” and “Glycine, serine, and threonine metabolism” with KEGG analysis of DEGs. **(D)** The relative mRNA expression of Chdh and Shmt with RNA-Seq (*n* = 4). **(E)** The intracellular level of glycine in M1 or M1-S (*n* = 6). **(F)** Production of IL-1β and TNF-α from TGPMs stimulated with LPS (1 μg/ml) plus IFN-γ (20 ng/ml) in completed medium, serine-depleted medium, or serine-depleted medium supplemented with formate (1.5 mM) and glycine (1.2 mM) or dNTPs (150 μM) (*n* = 4). Data are representative of two independent experiments. Data were analyzed by Mann-Whitney test and shown as the means ± SD. **(G)** The intracellular level GSH in TGPMs (*n* = 4). M1-S: thioglycolate-elicited peritoneal macrophages (TGPMs) were stimulated with LPS (1 μg/ml) plus IFN-γ (20 ng/ml) in serine deficiency medium; M1: TGPMs were stimulated with LPS (1 μg/ml) plus IFN-γ (20 ng/ml) in completed medium; M1+S: TGPMs were stimulated with LPS (1 μg/ml) plus IFN-γ (20 ng/ml) in medium with serine supplementation at dosage of 1.2 mM. Macrophages were stimulated with LPS plus IFN-γ for 15 h except indicated. Data were analyzed with one-way ANOVA **(F)** or unpaired t test **(D,E,G)** and represented as means ± SD except indicated. *P < 0.05, **P < 0.01, ***P < 0.001.

We then conducted RNA-Seq to further explore the influence of serine deprivation in M1 macrophages. Cluster analysis revealed that serine deprivation significantly affected the transcriptomic profile of M1 macrophages, in which 68 genes were up-regulated, while 108 genes were down-regulated (Figure 2A). The differently expressed genes (DEGs) were mostly enriched in biological process, including “multicellular organismal development,” “metabolic process,” and “cellular process” (Figure 2B, Supplementary Figure 1). KEGG-metabolic pathway analysis showed that serine deprivation affected the “biosynthesis of amino acids” and “glycine, serine, and threonine metabolism” (Figure 2C), especially down-regulated the expressions of *Chdh* and *Shmt* (serine hydroxymethyltransferase) (Figure 2D).

Serine could be metabolized by SHMT for the production of glycine to participate in one-carbon metabolism (34–36). Indeed, the intracellular level of glycine was lowered in M1 macrophages cultured with serine-depleted medium (Figure 2E). Then we supplemented formate, glycine, and/or dNTPs (the intermediates of one-carbon metabolism) in M1 macrophages cultured with serine-depleted medium. Interestingly, formate, glycine, dNTPs supplementation could not rescue the production of IL-1β and TNF-α from serine-deprived macrophages (Figure 2F). Recent compelling study has shown that serine supports IL-1β production from M1 macrophages through glycine-GSH axis (22). Unfortunately, serine deprivation had little effect on intracellular GSH level in M1 macrophages (Figure 2G). Collectively, serine deprivation alters the transcriptomic profile of M1 macrophages, especially glycine, serine and threonine metabolism, and supplementation of metabolites from one-carbon metabolism fails to rescue the production of cytokines from serine-deprived macrophages.

### Serine Deprivation Induces Metabolic Reprogramming in M1 Macrophages

**Figure 3:**
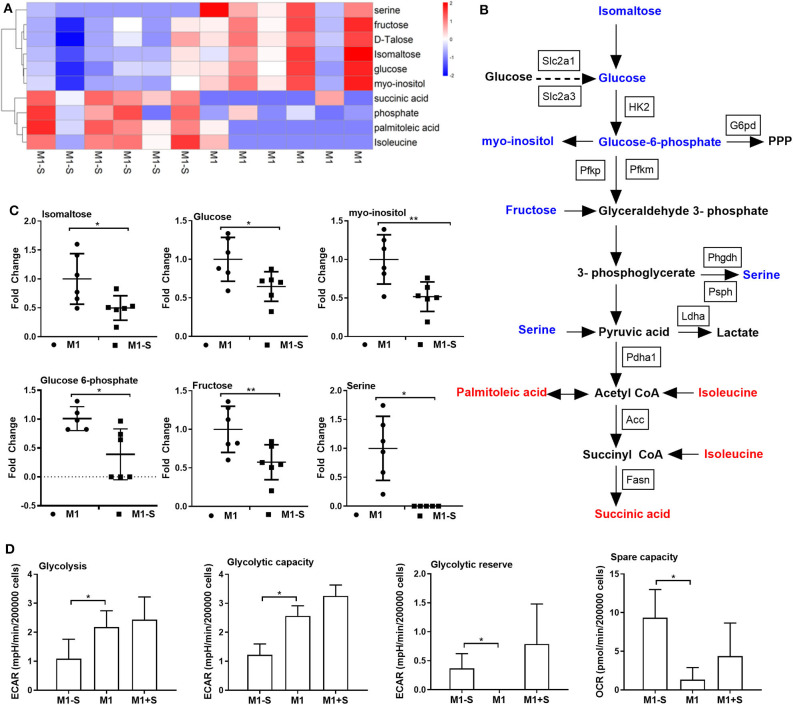
Serine deprivation results in metabolic reprogramming in M1 macrophages. **(A)** Heatmap analysis of different metabolites between M1 and M1-S after metabolomics analysis (*n* = 6). **(B)** The different metabolites in glycolysis after serine deprivation, decreased metabolites are in blue color, while increased metabolites are in red color (*n* = 6). **(C)** The fold change of different intracellular metabolites between M1 and M1-S (*n* = 6). Data were analyzed by unpaired *t*-test and shown as the means ± SD. **(D)** The extracellular acidification rate (glycolysis, glycolysis capacity and glycolysis reverse) and spare capacity of oxygen consumption rate among M1-S, M1, and M1+S (*n* = 4). M1-S: thioglycolate-elicited peritoneal macrophages (TGPMs) were stimulated with LPS (1 μg/ml) plus IFN-γ (20 ng/ml) in serine-deficient medium; M1: TGPMs were stimulated with LPS (1 μg/ml) plus IFN-γ (20 ng/ml) in completed medium; M1+S: TGPMs were stimulated with LPS (1 μg/ml) plus IFN-γ (20 ng/ml) in medium with serine supplementation at dosage of 1.2 mM. Macrophages were stimulated with LPS plus IFN-γ for 15 h except indicated. Data were analyzed with one-way ANOVA **(D)** or unpaired *t*-test **(C)** and represented as mean ± SD except indicated.*P < 0.05, **P < 0.01.

Given that M1 macrophage polarization shows notable metabolic alterations, and that serine deprivation significantly affects metabolism in M1 macrophages from RNA-Seq analysis; therefore, we performed metabolomics to assay the intracellular metabolites in M1 macrophages. The intracellular metabolites in M1 macrophages cultured with serine-depleted or serine-supplemented medium were obviously different from those in M1 macrophages cultured with normal medium (Supplementary Figures 2A-C; Supplementary Figures 3A-C). Similarly, the differential metabolites also could be enriched in glycine, serine, and threonine metabolism (Supplementary Figure 2D). The top significant differential metabolites were enriched in glycolysis, and most metabolites in glycolysis (e.g., isomaltose, glucose, myo-inositol, glucose 6-phosphate, and fructose) were down-regulated (Figures 3A–C), indicating serine deprivation restricts glycolysis of M1 macrophages. Notably, serine deprivation reduced the mRNA expression of *Pfkm* and *Pdha1* (glycolysis-related genes) in M1 macrophages, although it showed little effect on the expression of others (Supplementary Figures 2E-G). Therefore, we determined the ECAR and even OCR of M1 macrophages using a Seahorse extracellular flux analyzer. As expected, serine deprivation significantly blocked glycolysis of M1 macrophages (Figure 3D), while it had no significant effect on OXPHOS of M1 macrophages (Supplementary Figure 2H). Interestingly, serine supplementation had little effect on glycolysis and OCR (Figure 3D, Supplementary Figures 3D-F) in M1 macrophages.

**Figure 4:**
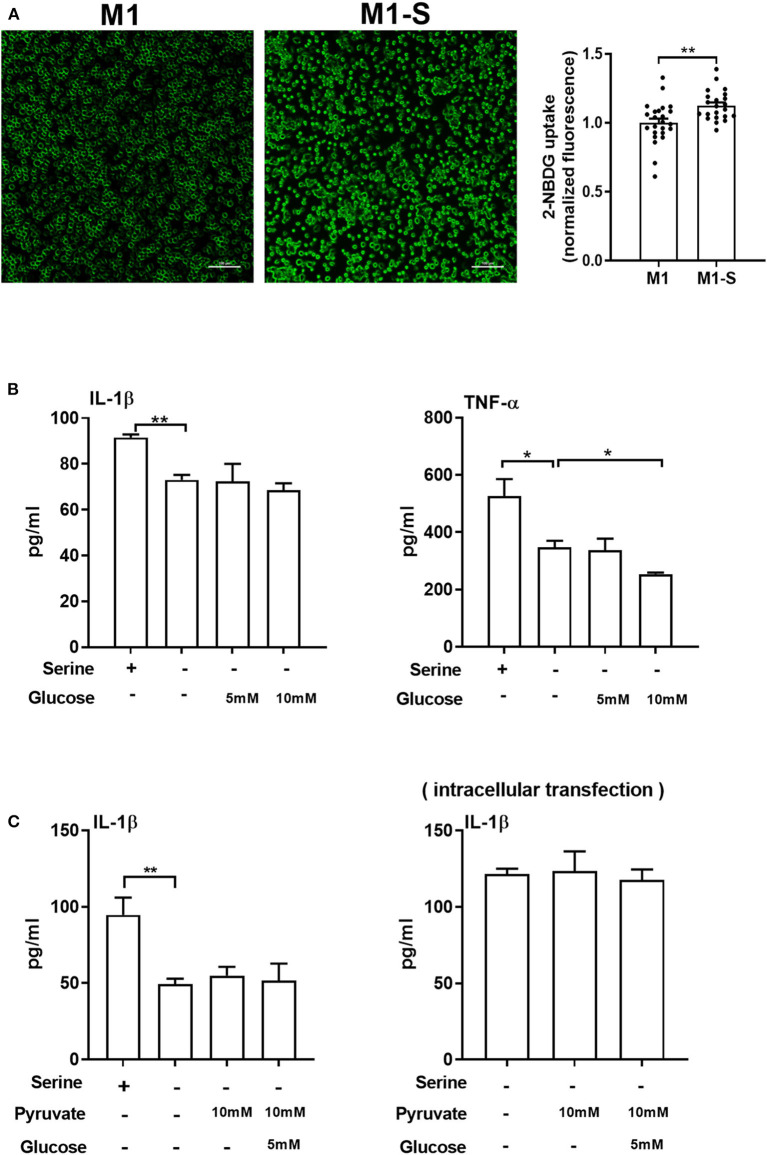
Serine deprivation promotes glucose uptake in M1 macrophages. **(A)** Glucose uptake in TGPMs with completed medium (M1) or serine deprived medium (M1-S) (*n* = 6). Data of two independent experiments were analyzed with unpaired *t*-test and represented as means ± SEM. **(B)** The secretion of IL-1β and TNF-α from TGPMs culturing in completed medium, serine deprived medium, serine deprived medium supplemented with extra glucose (5/10mM) for 15 h (*n* = 3). Data are representative of two independent experiments. **(C)** The secretion of IL-1β from TGPMs culturing in completed medium, serine deprived medium, serine deprivation medium with extracellular supplementation (Left) or intracellular transfection (Right) with sodium pyruvate (10 mM) alone or combined with glucose (5 mM) for 15 h (*n* = 3–4). Data are representative of two independent experiments. Thioglycolate-elicited peritoneal macrophages (TGPMs) were stimulated with LPS (1 μg/ml) plus IFN-γ (20 ng/ml) for 15 h except as indicated. Data were analyzed with unpaired *t*-test **(A)** or with one-way ANOVA **(B,C)** and represented as mean ± SD except indicated. *P < 0.05, **P < 0.01.

Although serine deprivation inhibited glycolysis in M1 macrophages, serine deprivation increased glucose uptake in M1 macrophages (Figure 4A), indicating that M1 macrophages cultured with serine-deleted medium increase their glucose uptake to fulfill bioenergetic demands, like synthesis of serine from 3-phosphoglycerate. However, addition of extra glucose could not rescue the production of IL-1β and TNF-α in M1 macrophages under serine deprivation (Figure 4B). Also, both extracellular supplementation and intracellular transfection with sodium pyruvate alone or combined with glucose were not capable of reversing the reduced IL-1β production in macrophages with serine deprivation (Figure 4C). Collectively, serine deprivation inhibits glycolysis in M1 macrophages, and addition of pyruvate or glucose cannot rescue the production of cytokines from serine-deprived macrophages.

### mTOR Signaling Contributes to IL-1β Production in Macrophages Under Serine Deprivation

**Figure 5:**
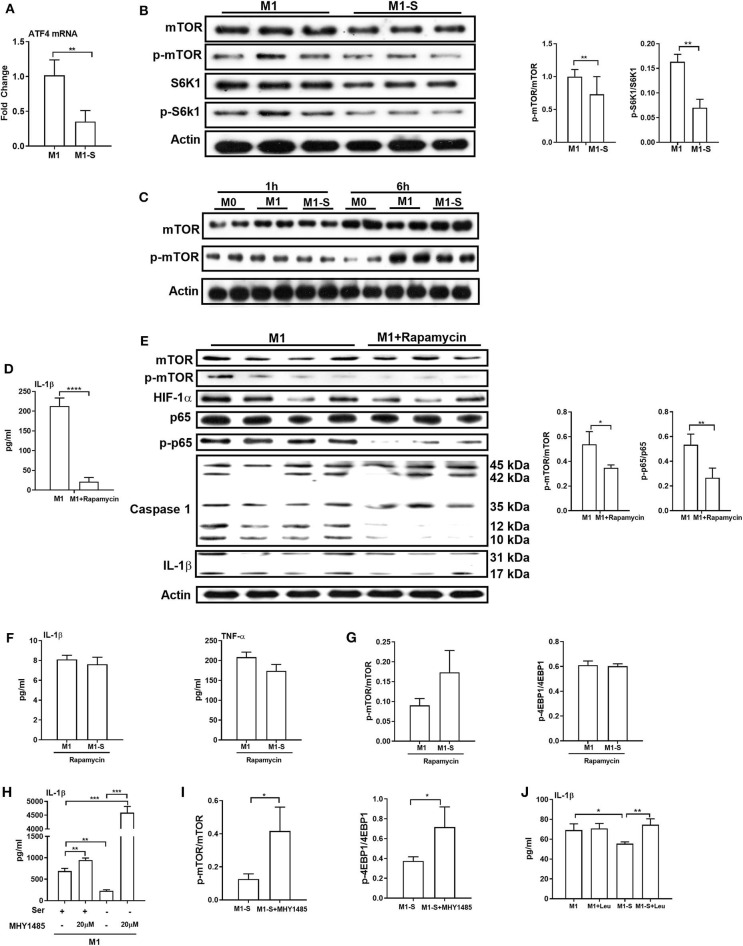
mTOR signaling supports IL-1β production in macrophages. The mRNA expression of ATF4 (*n* = 4), Data are represented as mean ± SEM. **(B)** Left: Immunoblotting to detect the protein abundance of mTOR, p-mTOR, S6K1, and p-S6K1 in TGPMs (*n* = 3); Right: Statistically analysis the relative abundance of proteins between two groups. Data are representative of three independent experiments. **(C)** Representative western bolt displaying time-dependent change of protein abundance of mTOR and p-mTOR in TGPMs (*n* = 4). **(D)** The secretion of IL-1β from TGPMs in completed medium and completed medium treated with Rapamycin (10μM) (*n* = 3). **(E)** Left: Immunoblotting to detect the protein abundance of mTOR, p-mTOR, HIF-1α, p65, p-p65, Caspase1, and IL-1β; Right: Statistically analysis the relative abundance of proteins between two groups (*n* = 3/4). **(F)** The secretion of IL-1β and TNF-α from TGPMs in M1 or M1-S group treated with Rapamycin (10 μM) for 15h (*n* = 3). **(G)** The relative abundance of p-mTOR/mTOR and p-4EBP1/4EBP1 in M1 or M1-S group treated with Rapamycin (10 μM) (*n* = 3). **(H)** The secretion of IL-1β from TGPMs in M1 or M1-S group treated with MHY1485 (20 μM) for 15 h or not (*n* = 3). **(I)** The relative abundance of p-mTOR/mTOR and p-4EBP1/4EBP1 in M1-S or M1-S group treated with MHY1485 (20 μM) (*n* = 3). **(J)** The secretion of IL-1β from TGPMs in M1 or M1-S group treated with Leucine (50 μM) for 15 h or not (*n* = 4). M1-S: thioglycolate-elicited peritoneal macrophages (TGPMs) were stimulated with LPS (1 μg/ml) plus IFN-γ (20 ng/ml) in serine deficiency medium; M1: TGPMs were stimulated with LPS (1 μg/ml) plus IFN-γ (20 ng/ml) in completed medium. Macrophages were stimulated with LPS plus IFN-γ for 15 h except indicated. Data were analyzed with one-way ANOVA **(H,J)** or unpaired *t*-test **(A,B,D–G,I)** and represented as mean ± SD except indicated. *P < 0.05, **P < 0.01, ***P < 0.001, ****P < 0.0001.

We next explored the mechanism by which serine deprivation suppresses IL-1β production in M1 macrophages. We firstly detected the mRNA expression of the transcription factor ATF4 as amino acid limitation activates the general control non-derepressible 2 (GCN2), which inhibits the general protein synthesis, but induces the translation of ATF4 (37). The mRNA expression of ATF4 was lowered in macrophages under serine deprivation (Figure 5A), ruling out that serine deprivation suppressed IL-1β production from M1 macrophages by affecting GCN2 signaling. The mTOR signaling is associated with M1 macrophage function and could be affected by alterations of amino acids (24, 38, 39). Serine deprivation significantly blocked mTOR activity based on the lower ratio of p-mTOR/mTOR and p-S6K1/S6K1 (Figure 5B), and even at 6 h post treatment (Figure 5C). Inhibition of mTOR signaling with rapamycin, like serine deprivation, significantly reduced IL-1β production and activation of p65 and Caspase-1 in M1 macrophages (Figures 5D,E). Also, inhibition of mTOR signaling with rapamycin blocked the effect of serine deprivation on the production of IL-1β and TNF-α in macrophages (Figures 5F,G). Notably, activation of mTOR signaling with MHY1485 (40–43) rescued the IL-1β production in macrophages cultured with serine-depleted medium (Figures 5H,I). Intriguingly, leucine, another mTOR activator (44, 45), also reversed the inhibition of IL-1β production in M1 macrophages under serine deprivation (Figure 5J). Collectively, these results indicate that serine deprivation blunts IL-1β production in M1 macrophages through blocking mTOR signaling.

### Lack of Serine Attenuates the Inflammatory Responses *in vivo*

**Figure 6:**
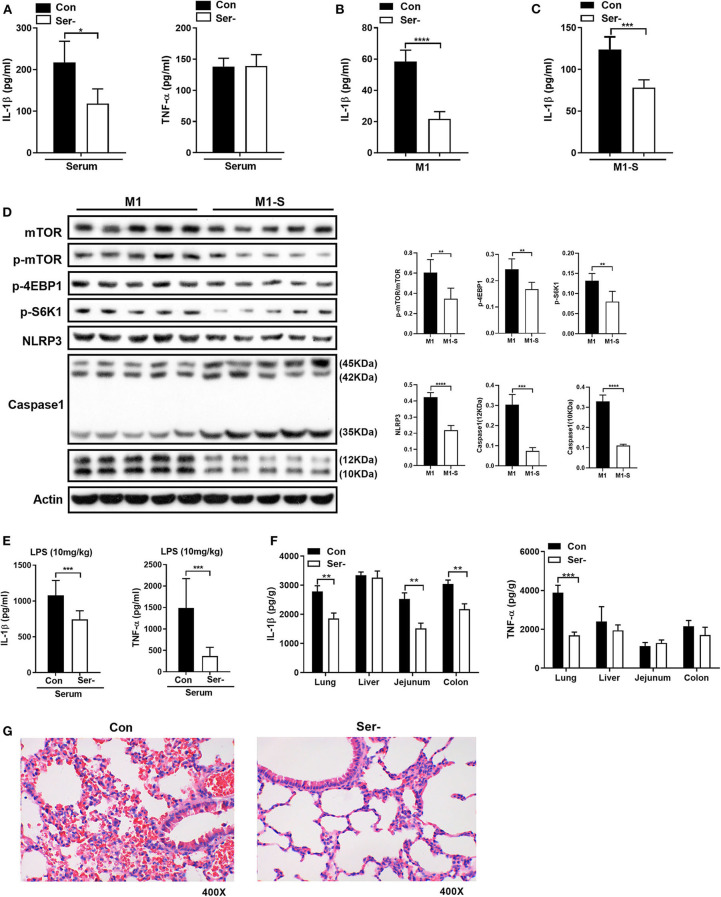
Serine-free diet reduces the mouse inflammatory responses *in vivo*. **(A)** The level of IL-1β and TNF-α in the serum of mice with control diet and serine-free diet (*n* = 8). **(B)** The secretion of IL-1β from TGPMs with control diet and serine-free diet in M1 condition (*n* = 5). **(C)** The secretion of IL-1β from TGPMs with control diet and serine-free diet in M1-S condition (*n* = 5). **(D)** Immunoblotting to detect the protein abundance of mTOR, p-mTOR, p-4EBP1, p-S6K1, NLRP3, and Caspase1 in TGPMs (*n* = 5). **(E)** The levels of IL-1β and TNF-α in the serum at 15 h post LPS challenge (10 mg/kg body weight) (*n* = 8). **(F)** The levels of IL-1β and TNF-α in the lung, liver, jejunum and colon at 15 h post LPS challenge (10 mg/kg body weight) (*n* = 8). **(G)** H&E staining to analyze the lung inflammation in mice with control diet (*n* = 9) or serine-free diet (*n* = 7) at 15 h post LPS challenge (10 mg/kg body weight). M1-S: thioglycolate-elicited peritoneal macrophages (TGPMs) were stimulated with LPS (1 μg/ml) plus IFN-γ (20 ng/ml) in serine deficiency medium; M1: TGPMs were stimulated with LPS (1 μg/ml) plus IFN-γ (20 ng/ml) in completed medium. Macrophages were stimulated with LPS plus IFN-γ for 15 h except indicated. Data were analyzed with unpaired *t*-test and represented as means ± SD except indicated. *P < 0.05, **P < 0.01, ***P < 0.001, ****P < 0.0001.

To ask whether serine deprivation could alleviate inflammation *in vivo*, 3-week-old mice were fed with serine-deprived feed (Supplementary Figure 4A) for 4 weeks. Both the weight gain and relative weight gain of mice fed with serine-deprived feed were lowered (Supplementary Figures 4B-C). Serine deprivation also reduced the serum level of IL-1β (Figure 6A). The TGPMs from these mice were then isolated and polarized into M1 phenotypes for further analysis. The production of IL-1β from M1 macrophages of mice fed serine-deprived diet were significantly lowered under serine-depleted or complete medium (Figures 6B,C) than those mice with control diet. M1 macrophages from mice with serine-deprived diet also had lower activation of mTOR signaling and inflammasome (i.e., NLRP3 and Caspase-1), than those from mice with control diet (Figure 6D).

After intraperitoneal injection of LPS (10 mg/kg), mice fed with serine-deprived diet had lower levels of IL-1β and TNF-α in serum (Figure 6E). Also, the levels of IL-1β and TNF-α in peripheral organs (lung in particular) of mice with serine-deprived diet were also lower than those mice with control diet (Figure 6F). H&E staining also showed that there was notable attenuation of hemorrhage in the lung of mice fed with serine-free diet after LPS challenge (Figure 6G), although no significant difference in the number of lung macrophages (Supplementary Figure 4D). Taken together, serine deprivation lowers inflammatory responses *in vivo*.

## Discussion

Macrophages show high diversity and can polarize into different phenotypes, including M1 and M2 macrophages (46, 47). M1 macrophages are crucial macrophage subsets to identify initial infections and injuries, however, deregulated activation of M1 macrophages is associated with various inflammatory diseases. Thus, there is increasing interesting in precise regulation of macrophage polarization, especially through cellular metabolic reprogramming (4, 8). Indeed, M1 macrophages have an enhanced glycolytic metabolism and impaired OXPHOS (48, 49). The swift upregulation of glycolytic metabolism in M1 macrophages not only produces ATP as energy to sustain their high secretory and phagocytic functions, but also feeds the pentose phosphate pathway (PPP) to fulfill nucleotide synthesis (50). Additionally, M1 macrophages also reprogram intracellular amino acid metabolism (e.g., glutamine) to exert their functions (51–55). Serine is a non-essential amino acid that used for the one-carbon metabolism, which supports nucleotide, SAM, NADPH, and GSH synthesis (30). Previous studies have shown that serine-supported one-carbon units are necessary for nucleotide production in cancer cells and lymphocytes to support their proliferation (15). Notably, LPS stimulation in macrophages enhances the serine synthesis and one-carbon metabolism (56), suggesting serine metabolism has critical importance for macrophage function; however, we are still lack of evidence to demonstrate this hypothesis. Currently, similar with a recent investigation (22), serine deprivation lowers the macrophage IL-1β. Interestingly, serine deprivation significantly down-regulates mRNA expression of SHMT, and lowers intracellular level of glycine. However, addition of formate (a metabolite in one-carbon metabolism for purine nucleotide biosynthesis) fails to rescue IL-1β mRNA expression from macrophages absence of serine (22), ruling out the possibility that serine mediates macrophage IL-1β production through one-carbon metabolism. In this study, addition of formate, glycine, formate plus glycine or dNTPs also cannot rescue the production of IL-1β from M1 macrophages cultured without serine. These results indicate that serine deprivation lowers the production of IL-1β from M1 macrophages independent on one-carbon metabolism. However, glycine still exists during serine deprivation, and presence of glycine may be available for complete *de novo* purine biosynthesis, which could mask the function of formate (17). Previous study also demonstrated that serine deprivation lowers the GSH level in LPS-stimulated macrophages, and that serine mediates macrophage IL-1β production through GSH production (57, 58). However, serine deprivation has little effect on intracellular GSH level in this study. Besides this discrepancy, here we find that serine deprivation also lowers the mRNA expression and production of TNF-α, which differ from the previous observation that serine deprivation only inhibits the IL-1β mRNA expression, but not TNF-α. The reason for these differences needs further investigation, and the differences in stimulus and the time for treatment may be involved.

This study also demonstrated that serine deprivation blocks glycolysis in M1 macrophages. Thus, serine deprivation may inhibit the IL-1β production in macrophages involving the glycolysis as serine can also be endogenously synthesized from glucose (59). Previous study has found that the inhibition of Phdgh (the rate-limiting step of serine biosynthesis from glucose) in CD8^+^ T cells reduces the production of serine from glucose, and suppresses the Teff cell proliferation (59). However, in this study, serine deprivation does not affect the expressions of Phgdh and Psph in M1 macrophages, and addition of different dosages of glucose or intracellular transfection with pyruvate alone or combined with glucose are unable to restore IL-1β production from macrophages under serine deprivation. Similarly, although inhibition of PHGDH with its inhibitor blocks the *de novo* synthesis of serine from glucose, this inhibition does not affect IL-1β expression in M1 macrophages (22). These results are suggesting that serine deprivation inhibits the IL-1β production in macrophages independent of its inhibition in the glycolysis. However, it is interesting to know the underlying mechanism by which serine deletion in the medium inhibits the glycolysis in M1 macrophages. This inhibition could not result from the defect in glucose transportation because serine deprivation promotes the glucose transportation. This study finds that serine deprivation lowers the mRNA expressions of Pfkm and Pdha1, however, the activities for these two enzymes need further validation, and the underlying mechanism by which serine deprivation lowers the mRNA expressions of Pfkm and Pdha1 in M1 macrophages needs further exploration.

Upon amino acid deprivation, GCN2 kinase is activated to phosphorylate eukaryotic initiation factor 2α (eIF2α) for suppressing global protein translation, which could ensure the normal growth and development of cells, and maintain physiological functions (60). Paradoxically, during this integrated stress response, translation initiation can still occur at alternative open reading frames and present in certain genes, such as ATF4 (37), leading to the up-expression of this factor. Surprisingly, our results indicate that serine deprivation down-regulates the mRNA expression of ATF4. Thus, it is not possible that serine deprivation restricts IL-1β production in M1 macrophages through GCN2 signaling. However, the activation of GCN2 signaling in M1 macrophages during serine deprivation needs further study. Accumulating studies have shown that mTOR senses the state of cellular energy and regulates its downstream signaling pathways to regulate cell metabolism, cell cycle progression and cell growth. Notably, M1 macrophages has higher activation of mTOR signaling than those in naïve macrophages (46). Here, M1 macrophages cultured with serine-depleted medium show reduction in mTOR signaling, and inhibition of mTOR signaling with rapamycin lowers the IL-1β production in M1 macrophages. Importantly, activation of mTOR signaling can rescue the IL-1β production in M1 macrophages under serine deprivation. These results strongly suggest that serine deprivation inhibits IL-1β production in M1 macrophages through mTOR signaling. Previously, we have shown that gamma-aminobutyric acid affects the activation of mTOR signaling in Th17 cells through its receptor in the cell membrane (61, 62), while others (e.g., leucine and arginine) modulates the activation of mTOR signaling in the cytoplasm (63, 64). Other amino acids, in particular leucine, have been shown to regulate cell differentiation and function through the mTOR signaling. mTOR activity, as reflected by ribosomal protein S6 phosphorylation, was partially inhibited by leucine restriction (44). In addition, it has been reported that arginine activates the mTORC1 pathway by binding with CASTOR1(the arginine sensor) (64). However, it remains to know the exact mechanism by which serine deprivation inhibits the mTOR signaling. Furthermore, adenosine 5′-monophosphate (AMP)-activated protein kinase (AMPK) activity inhibits mTOR signaling and induces autophagy, which could orchestrate macrophage polarization and contribute to macrophage functional plasticity (46, 65); thus it is interesting to further investigate whether serine deprivation affects AMPK activation and autophagy in M1 macrophages. In this study, serine deprivation also inhibits the activation of NF-κB and Caspases 1. It remains to investigate whether serine deprivation inhibits IL-1β production in M1 macrophages through these signaling pathways, and the mechanism by which serine deprivation inhibits these signaling pathways in M1 macrophages. Likewise, it is worthwhile to study whether serine metabolism could integrate these complicated cellular activities (e.g., intracellular signaling and epigenetic and metabolic reprogramming) to fine-tune macrophage polarization. However, it also should be kept in mind that different signaling mechanisms and complex amino acid metabolisms are engaged when individual amino acid is below or high than optimal concentration, resulting in the cellular signaling responses to amino acids are more complex than drug signaling.

Interestingly, serine deprivation *in vivo* reduces the serum level of IL-1β, and macrophages from mouse peritoneal cavity with serine-deprived diet have lower production of IL-1β, activation of mTOR signaling, and inflammasome. Notably, mice fed with serine-deprived diet have lower inflammation after intraperitoneal injection of LPS. As enhanced or prolong production of inflammatory cytokines from macrophages are associated with various inflammatory diseases, such as obesity, diabetes, and inflammatory bowel diseases (8), thus the results indicate an attractive strategy for modulating macrophage-associated diseases with serine-free diet. Although it is understandable and necessary to have *in vitro* study with standard medium for macrophages, the extrapolation of *in vitro* data presents many challenges, especially DMEM medium has higher concentration of glucose and serine than normal. It is interesting to know whether serine-free diet modulates the inflammatory responses through the intestinal microbiota. The intestinal microbiota affects numerous biological functions and is linked to the pathogenesis of various diseases (66, 67). We have shown that dietary melatonin supplementation modulates the host fatty acid metabolism and weanling stress through intestinal microbiota (68, 69). Thus, it is highly possible that serine-free diet modulates the inflammatory responses through intestinal microbiota. Indeed, serine is necessary for the growth potential of pathobiont-type Enterobacteriaceae, such as *Escherichia coli* LF82, in the inflammatory environment, and inflammation-induced *E. coli* LF82 are significantly blunted upon serine-free diet (70). Interestingly, the expansion of Enterobacteriaceae is a hallmark of Crohn's disease, and colonization by Enterobacteriaceae isolated from patients with Crohn's disease results in the development of severe colitis (71). Thus, the effect of serine-free diet on intestinal microbiota needs further investigations.

Taken together, our results demonstrate that serine deprivation inhibits M1 macrophage IL-1β production through mTOR signaling. This study highlights new regulatory mechanism by which serine tailors the immune responses of macrophages, implicating serine metabolism, and mTOR signaling as potential therapeutic targets for macrophage-associated diseases (e.g., cancer, obesity, and pathogen infection).

## Data Availability Statement

The raw data supporting the conclusions of this article will be made available by the authors, without undue reservation.

## Ethics Statement

The animal study was reviewed and approved by the Laboratory Animal Ethical Commission of the South China Agricultural University.

## Author Contributions

WR designed the experiment. SC, YX, FH, and JF conducted the experiment. SC, ZX, and BD analyzed the amino acids. LH and XZ helped the animal experiment. SC analyzed the data and prepared the figures. SC and YX drafted the manuscript. WR revised and approved the final manuscript. All authors contributed to the article and approved the submitted version.

## Conflict of Interest

The authors declare that the research was conducted in the absence of any commercial or financial relationships that could be construed as a potential conflict of interest.
