# Supplementary material for: Serine Supports IL-1β Production in Macrophages Through mTOR Signaling
Source: Front Immunol. 2020 Aug 27;11:1866. doi: 10.3389/fimmu.2020.01866 (PMC7481448; doi:10.3389/fimmu.2020.01866)
Supplement: Supplementary file 1 [file Data_Sheet_1.docx]

Supplementary Material

## Supplementary Figures

**
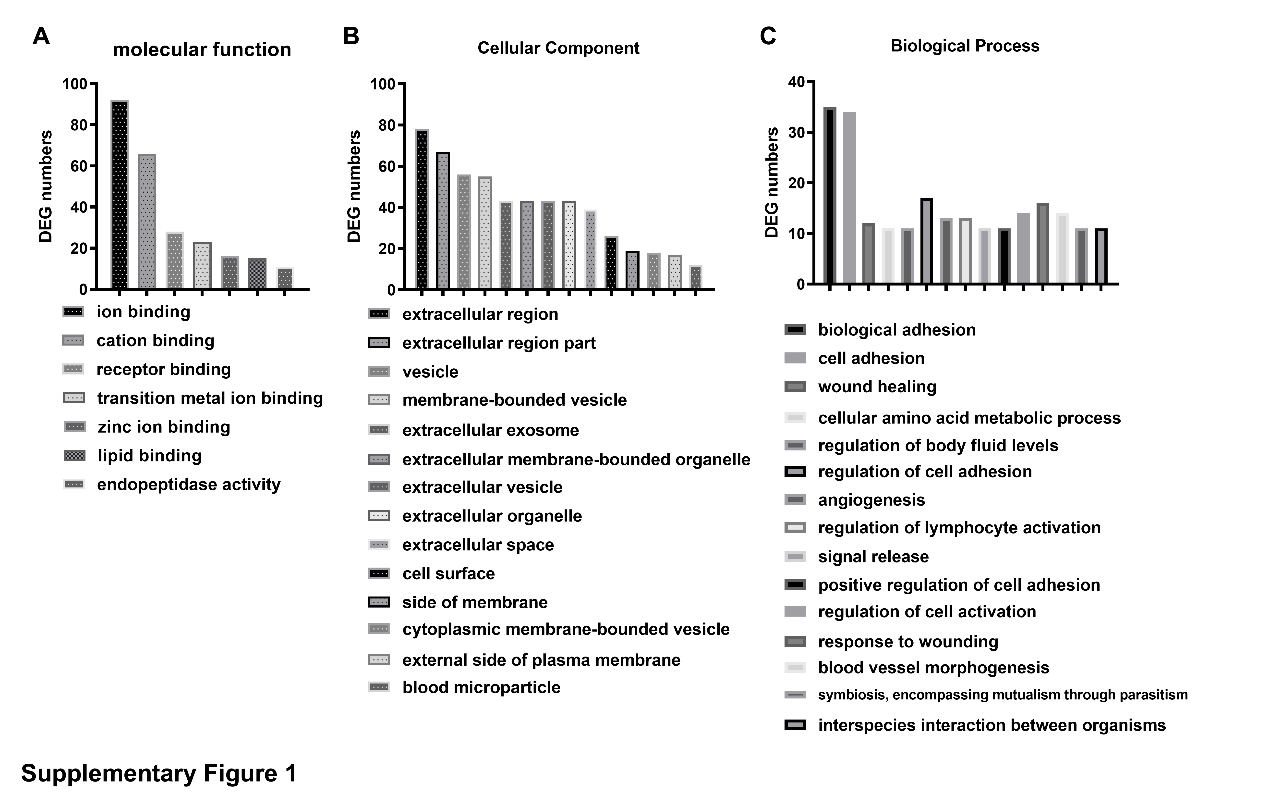
Supplementary figure1.**

**(A**). GO analysis of DEG numbers which enriched in molecular function (n=4).

**(B**). GO analysis of DEG numbers which enriched in cellular component (n=4).

**(C**). GO analysis of DEG numbers which enriched in biological process (n=4).

**
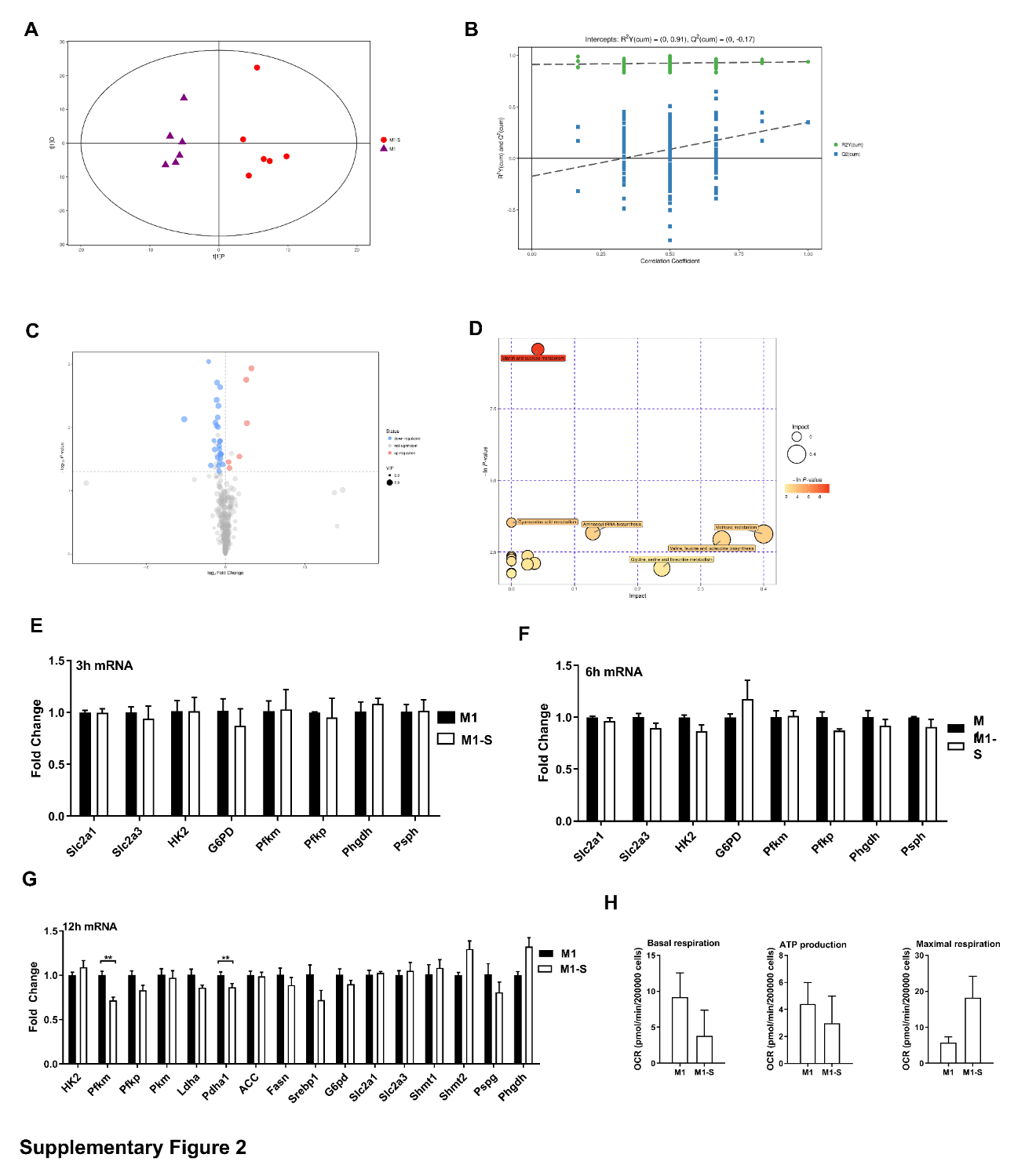
Supplementary figure2.**

**(A**). Principal Component Analysis (PCA) between M1 and M1-S group (n=6).

**(B**). OPLS-DA permutation plot between M1 and M1-S group (n=6).

**(C)**. Volcano plot between M1 and M1-S group with blue representing significantly down-regulated metabolites, up-regulated metabolites in red color, while those that were not significantly different were shown in gray.

**(D**). Metabolic pathway analysis about the significantly different metabolites between M1 and M1-S group (n=6).

**(E-G**). The effect of serine deprivation on the mRNA expressions of genes in glycolysis and TCA at different time points (3, 6 and 12 hours). Data are represented as mean ± SEM.

**(H**). The effect of serine deprivation on macrophages oxygen consumption rate (basal respiration, ATP production and maximal respiration) (n=4). Data are represented as mean ± SEM.

Data were analyzed with unpaired t test and represented as means ± SD except indicated. **P < 0.01.

**
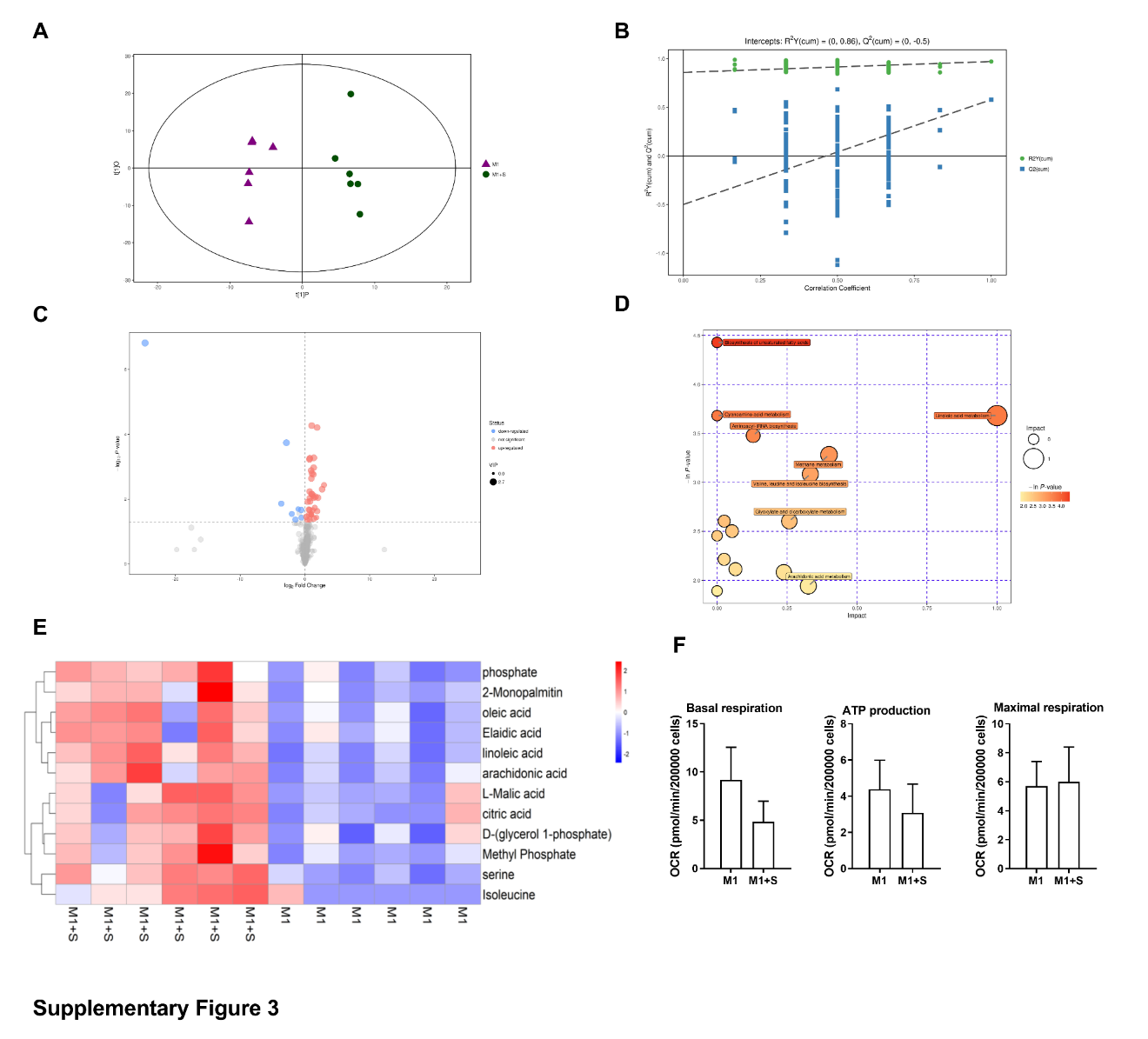
Supplementary figure3.**

**(A**). Principal Component Analysis (PCA) between M1 and M1+S group (n=6).

**(B**). OPLS-DA permutation plot between M1 and M1+S group (n=6).

**(C**). Volcano plot between M1 and M1+S group with blue representing significantly down-regulated metabolites, up-regulated metabolites in red color, while those that were not significantly different are shown in gray.

**(D**). Metabolic pathway analysis about the significantly different metabolites between M1 and M1+S group (n=6).

**(E**). Heatmap analysis of significantly different metabolites in macrophages between M1 and M1+S group (n=6).

**(F**). The effect of serine supplementation on macrophages oxygen consumption rate (basal respiration, ATP production and maximal respiration) (n=4). Data are represented as mean ± SEM.

Data were analyzed with unpaired t test and represented as means ± SD except indicated.

**
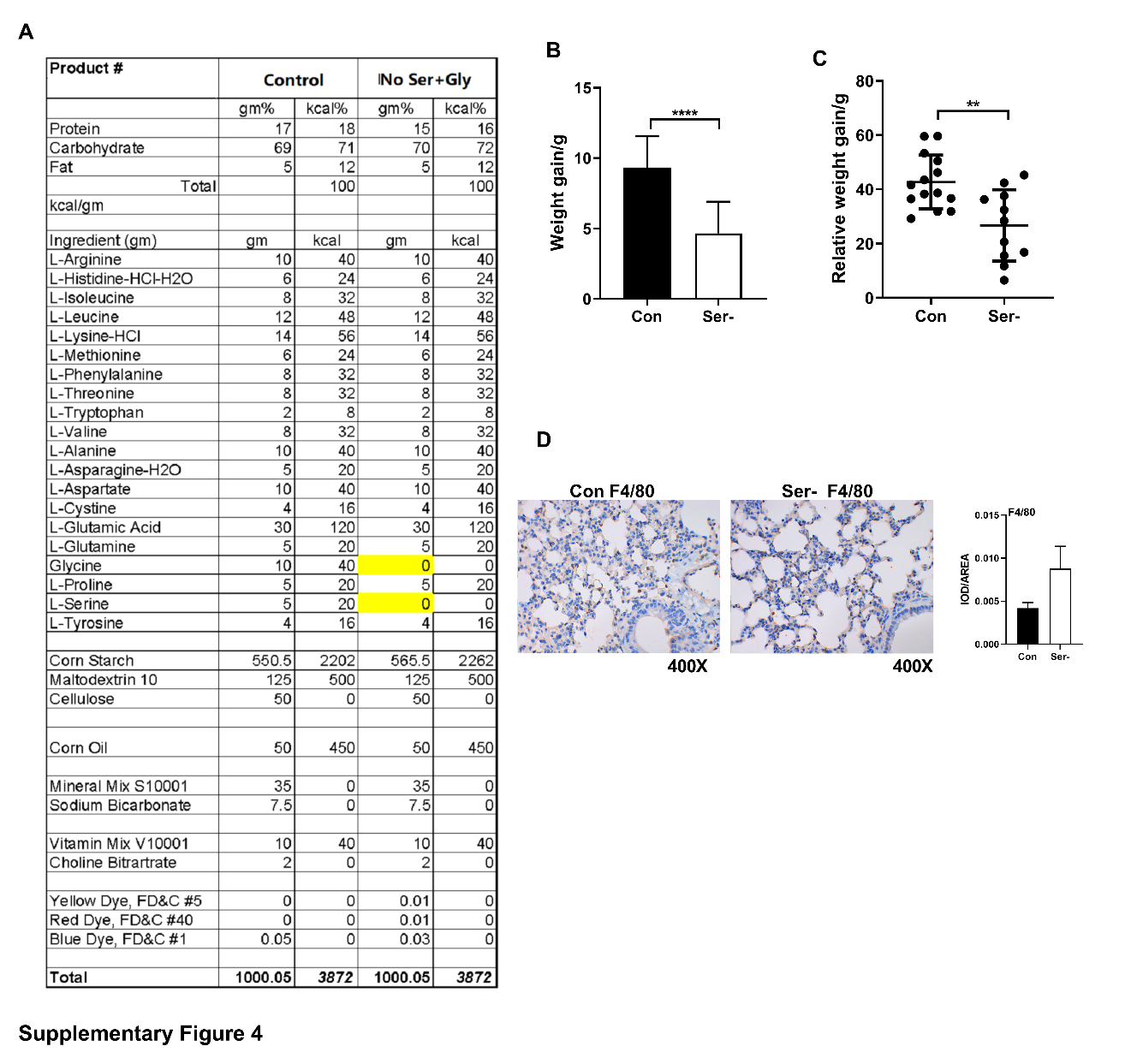
Supplementary figure4.**

**(A**). Feed formula table of serine-deprived feed.

**(B-C**). The weight gain and relative weight gain of mice fed with control feed (n=14) and serine-deprived feed(n=11).

**(D**). The lung abundance of F4/80 was determined by immunohistochemistry in mice with control diet (n=9) or serine free diet (n=7) under LPS challenge.

Data were analyzed with unpaired t test and represented as means ± SD. **P < 0.01, ****P < 0.0001.

**Supplementary Table 1**

| **Primer** | **Forward primer** | **Reverse primer** |
| --- | --- | --- |
| Mouse b-Actin | GTCCCTCACCCTCCCAAAAG | GCTGCCTCAACACCTCAACCC |
| Mouse TNF-α | ACGTCGTAGCAAACCACCAA | GCAGCCTTGTCCCTTGAAGA |
| Mouse ATF4 | GAAATGGCCGGCTATGG | TCCCGGAAAAGGCATCCT |
| Mouse Psph | AGGAAGCTCTTCTGTTCAGCG | GAGCCTCTGGACTTGATCCC |
| Mouse Phgdh | GGAGGCTTTCCAGTTCTGCT | CTGCGATCCCCTCTCCCTAT |
| Mouse Shmt1 | CAGCTCCCGAAACCAAGTGA | TGCTGTAAACCTCGGCATCA |
| Mouse Shmt2 | TTCACTCGAACTTCACGGGG | AGCTGACCACATCTCCGAGT |
| Mouse Gapdh | GTCGGTGTGAACGGATTTG | TAGACTCCACGACATACTCAGCA |
| Mouse Ldha | AAACCGAGTAATTGGAAGTGGTTG | TCTGGGTTAAGAGACTTCAGGGAG |
| Mouse Ldhb | GGACAAGTGGGTATGGCATGTG | CCGTCACCACCACAATCTTAGA |
| Mouse Pfkm | GATCTTTGCCAACACCCCTGACTC | GGTCGGAGGTGTCCAGATCAATCTC |
| Mouse Pfkp | GGTACAGATTCAGCCCTGCACC | GTCGGCACCGCAAGTCAAGG |
| Mouse Hk2 | CCGTGGTGGACAAGATAAGAGAGAACC | GGACACGTCACATTTCGGAGCCAG |
| Mouse Pdha1 | CTACAGGATGATGCAGACTGTGC | GAGGTGGTCCGTAGGGTTTATGC |
| Mouse Slc2a1 | CTGGACCTCAAACTTCATTGTGGG | GGGTGTCTTGTCACTTTGGCTGG |
| Mouse Slc2a3 | GCATTTGGCACACTAAACCA | GCCCAGAATAAAGTCCAAACC |
| Mouse ACC | ACCTGCCACAGAAACCATTC | CGTGCTGTAGCAAAAGTGGA |
| Mouse Fasn | CCTGGATAGCATTCCGAACCT | AGCACATCTCGAAGGCTACACA |
| Mouse Srebp1 | GGAGCCATGGATTGCACATT | CAGGAAGGCTTCCAGAGAGG |
| Mouse PKM | ATTACCAGCGACCCCACAGAA | ACGGCATCCTTACACAGCACA |
| Mouse G6pd | CTCCTGCAGATGTTGTGTCT | TCATTGGGCTGCATACGGA |
